# Apremilast therapy increases CD39^+^CD4^+^ T cells in peripheral blood of patients with psoriatic disease: a retrospective observational pilot study

**DOI:** 10.1007/s00296-026-06114-3

**Published:** 2026-05-06

**Authors:** Athanasios Mavropoulos, Sotirios G. Tsiogkas, Theodora Simopoulou, Efthimios Dardiotis, Efterpi Zafiriou, Lazaros I. Sakkas, Dimitrios P. Bogdanos

**Affiliations:** 1 Department of Rheumatology and Clinical Immunology, Faculty of Medicine, University of Thessaly, Larissa, Greece; 2 Department of Neurology, Faculty of Medicine, University of Thessaly, Larissa, Greece; 3 Department of Dermatology, Faculty of Medicine, University of Thessaly, Larissa, Greece; 4 IASO General Hospital, Larissa, Greece

**Keywords:** Arthritis, Psoriatic, Apremilast, Ectonucleoside Triphosphate Diphosphohydrolase 1 (CD39), FOXP3 protein, human, T-Lymphocytes, Regulatory, Psoriasis, Blood Cells, Mononuclear, Interleukin-10, Flow Cytometry

## Abstract

Psoriasis and psoriatic arthritis (PsA) are chronic inflammatory diseases, recognized as one entity, termed psoriatic disease (PsD). CD39 is an ectonucleotidase that hydrolyzes ATP to adenosine and is expressed on a human Treg sub-population capable of exerting interleukin (IL-) 17 suppression. We aimed to investigate the effect of apremilast on CD39 expression in T cells from patients with PsD. Peripheral blood mononuclear cells (PBMCs) were analyzed by multicolor flow cytometry and appropriate monoclonal antibodies. Intracellular cytokine production of PBMCs was measured after in vitro PMA plus ionomycin stimulation. At 6 weeks post-treatment**,** apremilast inhibited IL-17 and interferon-γ (IFNγ) production, increased IL-10 production in both CD4+T cells and CD4-T cells, and inhibited IL-6 production in CD4-T cells. At baseline, levels of CD4+CD39+ cells were decreased in patients compared to controls. At 6 weeks post-treatment, CD4+CD39+ cells increased. Furthermore, CD4+CD39+ cells producing IL-10 increased in patients who achieved a PASI response. A significant proportion of IL-10-producing CD39+ cells were CD56-CD11c- cells. CD4+FoxP3+ cells were decreased in patients compared to controls. At 6 weeks post-treatment CD4+FoxP3+ cells, and CD4+FoxP3+CD39+ cells increased, although CD4+CD25hiFoxP3+ Treg cells did not change significantly. If confirmed in larger studies, CD4+CD39+ cells producing IL-10 could likely become associated with the response to apremilast.

**Supplementary Information:**

The online version contains supplementary material available at 10.1007/s00296-026-06114-3.

## Introduction

T regulatory (Treg) cells control immune responses and prevent autoimmunity[1]. High levels of CD25 surface expression and forkhead box P3 (FoxP3) have been suggested as Treg signature markers. Another marker of Treg cells is CD39, an ectonucleotidase that hydrolyzes adenosine triphosphate (ATP) to adenosine (ADO), a potent immune suppressor that activates adenyl cyclase to catalyze ATP into cyclic adenosine monophosphate (cAMP) and inhibit T cell function[2]. A human Treg sub-population expressing CD39 is capable of exerting interleukin- (IL-) 17 suppression[3]. CD39 appears to sustain the suppressive ability of Treg cells, as CD39^hi^ Treg cells maintain stable FoxP3 expression under inflammatory conditions, whereas CD39^low^ Treg cells have been found to differentiate into T helper (Th)1 producing interferon (IFN) γ or Th17 cells producing IL-17 [4].

The Treg cell impairment, both functional and numerical, has been noted in psoriasis and psoriatic arthritis (PsA)[5], chronic inflammatory diseases considered to be one entity, the psoriatic disease (PsD)[6][7, 8]. Recent systematic reviews further emphasize the disturbed balance between tissue-resident memory T cells and Tregs as a key pathogenic feature of PsD[9]. In PsD, proinflammatory molecules induce the differentiation of T cells towards a Th17 phenotype [10].

CD39^+^ Treg cells, mainly FoxP3^+^ [11, 12], have been reported to suppress IFNγ and IL-17 in a cell-contactmanner[12, 13]. Interestingly, the CD39^+^ Treg subset has been found to co-express CCR6[11]. A functional deficit of CD39^+^ Treg cells has been reported in patients with multiple sclerosis (MS) [12].

Apremilast is a small molecule phosphodiesterase 4 (PDE4) inhibitor approved for the treatment of psoriasis and PsA. PDE4 decreases intracellular cAMP, thus promoting inflammatory molecule production, whereas apremilast increases cAMP levels and regulates inflammation through multiple cAMP downstream effectors important for IL-10 production [14]. Our group has previously reported that apremilast increased IL-10-producing regulatory B cells in patients with PsD [15], but the exact in vivo effect of apremilast on IL-10 producing T cells and in particular on CD39^+^ Treg cells remains unclear.

Our study aimed to investigate the effect of apremilast on CD39 expression in T cells from patients with PsD.

## Patients and methods

### Participants

This was a retrospective, observational pilot study based on biobanked peripheral blood mononuclear cell (PBMC) samples from patients with PsD treated with apremilast. All samples had been collected and stored originally under standardized conditions at the biobank of the Laboratory of Rheumatology and clinical Immunology, at the University Hospital of Larissa. The present analysis was conducted on de-identified specimens. Fifteen consecutive patients with disease (3 females, 12 males; mean age 51.5 years) followed up at the Outpatient Clinics of Rheumatology and Dermatology, University General Hospital of Larissa and 12 healthy controls (mean age 49.4 years) were included in the study. Of the 15 patients, 5 were diagnosed with PsA, 10 with psoriasis, 3 patients were treatment-naïve, and 13 were biologics-naïve Demographic and clinical characteristics are presented in Table 1. Clinical assessment included the psoriasis area, and severity index (PASI). Patients received apremilast 30 mg twice daily for at least 6 weeks. Blood samples were collected before treatment and at 6 weeks after apremilast initiation. This study was conducted and reported in accordance with the STROBE (Strengthening the Reporting of Observational Studies in Epidemiology) guidelines (EQUATOR Network), as shown in Appendix 1. All patients participating in the study have signed a written informed consent, in accordance with the revised Declaration of Helsinki. The study was approved by the Ethics Committee of the Scientific Council of the University General Hospital of Larissa (protocol 41636/01-10-19 & 20,931/11-06-2021).

**Table 1:** Clinical and demographical characteristics of enrolled patients

Patient ID	Age	Gender	Previous treatments	Baseline PASI	6 weeks PASI	PsA
1	55	F	Methotrexate	10.2	2.4	Yes
2	63	M	Methotrexate, acitretin	8.6	1.8	No
3	57	F	Methotrexate	12.6	2	No
4	52	M	Etanercept	20	5	Yes
5	50	M	–	9.2	2	No
6	68	M	Acitretin, cyclosporin	21.9	6.9	Yes
7	62	M	Cyclosporin	5.5	3.1	No
8	36	M	Methotrexate	4.5	1	No
9	65	M	Cyclosporin	21.9	10	Yes
10	45	M	–	3	0	No
11	42	F	Cyclosporin	2.3	3	No
12	62	M	Cyclosporin	12.4	9.1	No
13	32	M	Adalimumab	15.6	7	Yes
14	33	M	–	3.6	1	No
15	50	M	Methotrexate	2.9	2.9	No

*F* female, *M* male, *PSA* diagnosis of psoriatic arthritis, *PASI* psoriasis area severity index

### Cell isolation and cryopreservation

Peripheral blood samples from patients and controls were collected by venipuncture in preservative-free heparin tubes. PBMCs were isolated by conventional density gradient centrifugation described previously [15]. Specifically, blood aliquots were layered onto an equal volume of Ficoll-Hypaque (10 ml Lymphoprep™) density gradient solution (Axis-Shield, Oslo, Norway). PBMCs were isolated by centrifugation at 300 g, washed twice with RPMI-1640 (GIBCO™ -Thermo Fisher Scientific, Waltham, MA, USA), counted, and their viability, determined by trypan blue exclusion, routinely exceeded 95%. Cells were re-suspended in freezing medium containing 10% DMSO and 70% FCS, aliquoted into cryogenic vials (Corning™, Thermo Fisher Scientific), kept at − 80 °C for one day, and then stored in liquid nitrogen tanks until used. Upon thawing, PBMCs were washed with serum-free RPMI-1640, counted to confirm more than 95% cell viability, pelleted, and resuspended at 10^6^ cells/mL in RPMI culture medium supplemented with L-glutamine and 10% heat-inactivated fetal bovine serum (FBS) (Biosera Europe, Nuaille, France). PBMCs were seeded in 24-well plates and allowed to rest at 37 °C in a CO2 incubator for at least one hour before stimulation. Isolated PBMCs (0.5–1 × 10^6^ cells) were washed in PBS and re-suspended in staining buffer (PBS + 1% FBS + 0.09% sodium azide) prior to incubation with labeled MoAbs specific for cell surface antigens for 30 min on ice and subsequent fixation with paraformaldehyde (2%). Logarithmic amplification was used during data collection. At least 2 × 10^5^ events within the lymphocyte gate were collected to accurately measure infrequent cell subtypes.

### Phenotypic analysis of peripheral blood mononuclear cells by flow cytometry

Phenotypic assessment and enumeration of PBMCs was achieved with the following monoclonal antibodies (MoAbs): FITC-conjugated anti-CD3 (clone UCHT-1); PE-, and PE-Cy7-conjugated anti-CD56 (clones C5.9 and B159); PE-, APC-, and PE-Cy7-conjugated anti-CD4 (clone RPA-T4); FITC- and APC-conjugated anti-CD19 (clones HIB19 and HD37); FITC-conjugated anti-CD7 (clone 4H9); FITC- and PE-Cy7-conjugated anti-CD39 (clone A1); PE-conjugated anti-CD11c (clone B-ly6); PE-conjugated anti-CD25 (clone BC96). All MoAbs were obtained from BD Biosciences (Mountain View, CA, USA), BioLegend (San Diego, CA, USA) and Merck-Millipore (Burlington, MA, USA). Flow cytometric analysis was performed in Guava® EasyCyte8 (Merck-Millipore, Burlington, MA, USA) benchtop flow cytometer.

### Intracellular cytokine production by peripheral blood cell subsets:

PBMCs were left untreated or cultured in 10% RPMI supplemented with phorbol 12-myristate 13-acetate (PMA) plus ionomycin in the presence of brefeldin A for 5 h. Phorbol 12-myristate 13-acetate (PMA) and ionomycin were obtained from Sigma-Aldrich-Merck (Gillingham, UK) and were used at 50 μg/ml and 1 μg/ml, respectively, to stimulate PBMCs non-specifically. Brefeldin A (Cayman chemical) was used at a final concentration of 10 μg/ml to block cytokine secretion. Cells were surface-stained, fixed, and permeabilized using commercially available Perm/Wash buffers (BD Biosciences).

Intracellular cytokines were detected using the following MoAbs: APC-conjugated anti-IFNγ (clone 4S.B3), and PE-conjugated anti-IL-17 (clones BL-168), PE-conjugated anti-IL-6 (clone MQ2-13A5) PE- and APC-conjugated anti-IL-10 (clone JES3-19F1).

### T regulatory cells

CD4^+^CD25^hi^FoxP3^+^ Treg cells were identified using BioLegend's True-Nuclear™ Transcription Factor Buffer Set per manufacturer's guidelines. In brief, human peripheral blood lymphocytes were initially surface stained with APC-conjugated anti-CD4 and PE-conjugated anti-CD25 MoAbs and then treated with True-Nuclear™ Transcription Factor Buffer Set. Cells were then stained with FITC-conjugated anti-human FoxP3 MoAb (clone 206D).

### Statistical analysis

Percentages of cells expressing cell surface markers and mean fluorescence intensities (MFI) were described as medians of the individuals in each group. Variation in each patient group was defined by standard deviation (SD). Differences between healthy controls and patients and between patient groups were tested, following a Shapiro–Wilk test. Depending on distribution, group differences were analyzed using either a two-tailed Student’s *t*-test or the non-parametric Mann–Whitney *U*-test. *P*-values ≤ 0.05 were considered statistically significant. For paired data that did not follow a normal distribution, we calculated Hodges Lehmann median differences along with 95 percent confidence intervals and rank-biserial effect sizes marked as r. When tied observations were present, asymptotic *p*-values were reported. All statistical calculations were performed with Graph Pad Prism and R software.

## Results

At 6 weeks, apremilast improved skin manifestations and the mean PASI decreased from 10.3 to 3.8. Among 15 PsD patients, 7 patients achieved PASI75 while 11 patients achieved PASI50.

### Apremilast inhibited proinflammatory cytokine production

**Fig. 1:**
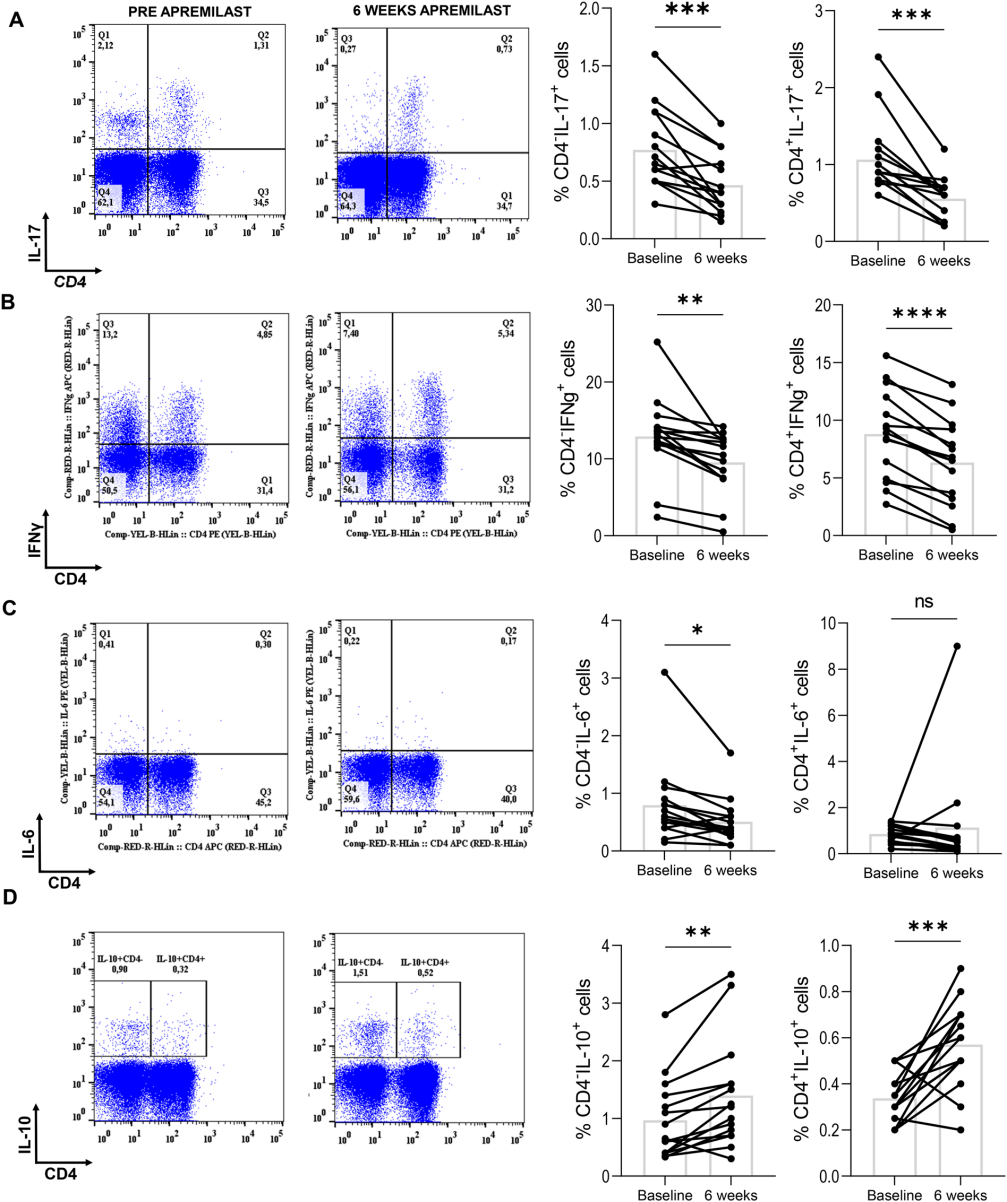
Apremilast inhibited proinflammatory cytokines and increased interleukin (IL-) 10. Peripheral blood mononuclear cells (PBMCs) were left untreated or cultured in 10% RPMI culture medium supplemented with phorbol 12-myristate 13-acetate (PMA) plus ionomycin in the presence of brefeldin A for 5 h. Cells were surface stained, fixed and subsequently permeabilized. Intracellular staining followed. Representative flow cytometry plots and graphs presenting the in vivo apremilast-mediated changes of **a** IL-17, **b** interferon (IFN) γ, **c** IL-6, and **d** IL-10. Paired t-tests were executed. A p-score < 0.05 was considered significant. Graphs are depicting mean ± SD; ns p > 0.05; * p ≤ 0.05; ** p ≤ 0.01; *** p ≤ 0.001; **** p ≤ 0.0001

We initially assessed the effect of in vivo apremilast administration in stimulated cells' ex vivo cytokine production. IL-17 and IFNγ have been found to participate in the development of an inflammatory environment in the skin and joint[16]. At baseline, IL-17 and IFNγ were elevated in patients compared to controls but at 6 weeks post-apremilast treatment, production of both cytokines was significantly decreased. The inhibitory effect of apremilast was observed in both CD4^+^ T cells (IL-17: median pair differences [IQR] 0.45 [0.2 to 0.65], Hodges-Lehmann 95% CI 0.30 to 0.70, p = 0.0007, r = 0.88; IFNγ: median pair differences [IQR] 2.45 [1.85 to 3.14], Hodges-Lehmann 95% CI 1.85 to 3.15, p = 0.00006, r = 0.88) and CD4^−^ T cells (IL-17: median pair differences [IQR] 0.30 [0.15 to 0.44], Hodges-Lehmann 95% CI 0.18 to 0.44, p = 0.0009, r = 0.87; IFNγ: median pair differences [IQR] 2.82 [1.5 to 4.35], Hodges-Lehmann 95% CI 1.60 to 4.65, p = 0.00006, r = 0.88). We also examined IL-6 production, as IL-6 induces Th17 development and inhibits Treg differentiation[17]. IL-6 production by CD4^−^T cells was decreased (median pair differences [IQR] 0.25 [0.15 to 0.375], Hodges-Lehmann 95% CI 0.125 to 0.40, p = 0.0041, r = 0.75) post apremilast treatment (Fig. 1).

### Apremilast increased IL-10 production

**Fig. 2:**
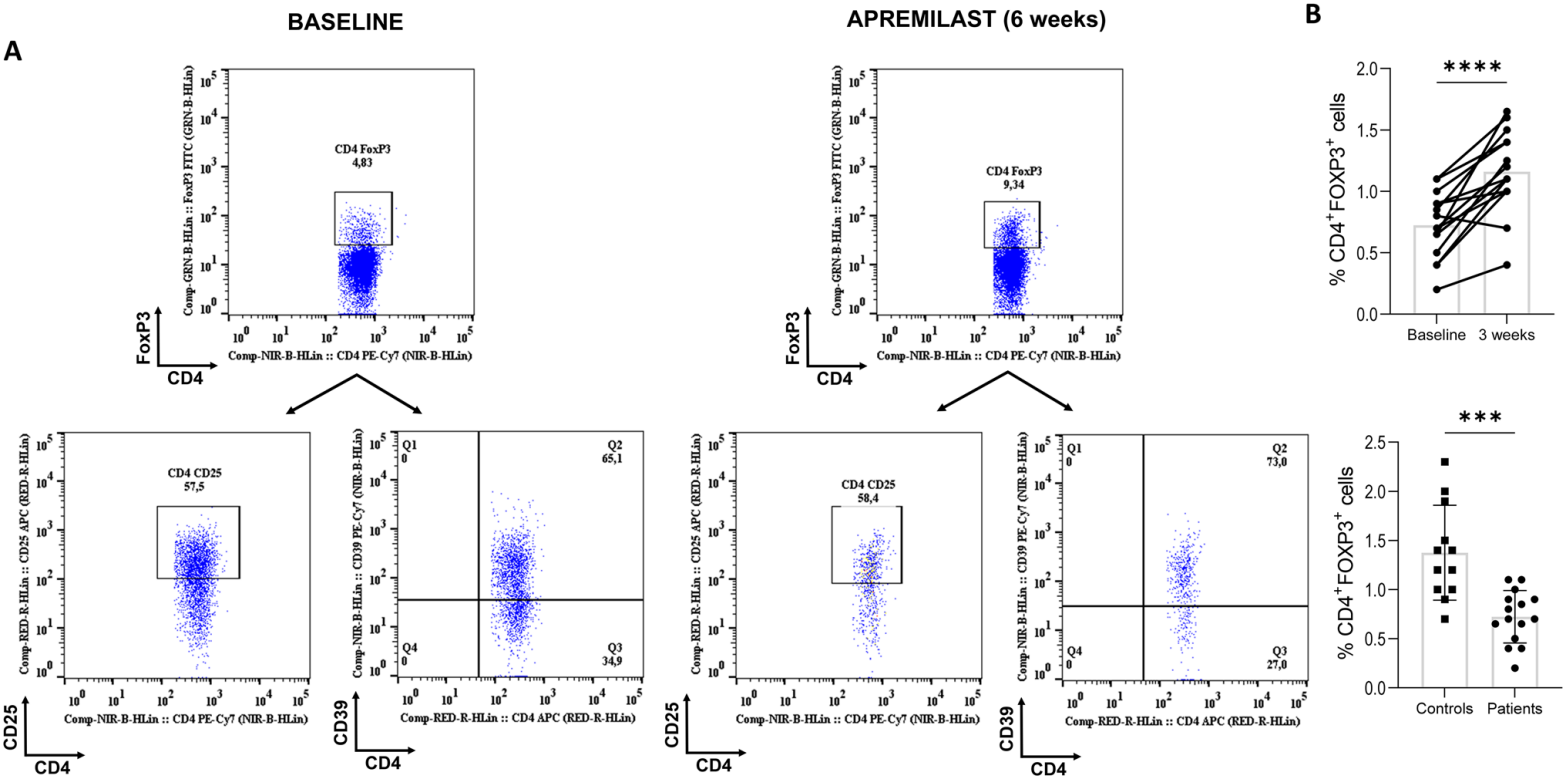
Apremilast did not affect T regulatory cell percentages, but increased (forkhead box P3) FoxP3 expression.** a** Flow cytometry plots illustrating that proportions of T regulatory cells remained largely unchanged after therapy, but FoxP3^+^ T helper (Th) cells significantly increased. **b** Graphs presenting increased levels of FoxP3^+^ Th cells in periphery of controls compared to patients and increased levels of FoxP3^+^ Th cells after therapy. Unpaired and paired t-tests were executed. A p-score < 0.05 was considered significant. Graphs are depicting mean ± SD; *** p ≤ 0.001; **** p ≤ 0.0001

Apremilast treatment significantly increased IL-10 production. The increase was noted in both CD4^+^T cells and CD4^−^ cells (CD4^+^: median pair differences [IQR] − 0.25 [− 0.35 to − 0.15], Hodges-Lehmann 95% CI − 0.35 to − 0.13, p = 0.0031, r = 0.772; CD4^−^: median pair differences [IQR] − 0.40 [− 0.6 to − 0.175], Hodges-Lehmann 95% CI − 0.60 to − 0.20, p = 0.003, r = 0.777). To better understand the effect of apremilast on IL-10 expression on T cells, we assessed Treg cell markers (Fig. 2). FoxP3 expression was decreased in CD4^+^T cells in patients compared to controls (− 0.65, 95% CI − 0.95 to − 0.35, p = 0.0001). At 6 weeks post treatment, FoxP3 expression was significantly increased (median pair differences [IQR] − 0.45 [− 0.625 to − 0.25], Hodges-Lehmann 95% CI − 0.60 to − 0.275, p = 0.0011, r = 0.851), whereas Treg cells (CD4^+^CD25^hi^FoxP3^+^) did not significantly change.

### Apremilast increased CD4^+^CD39^+^ cells and enriched CD39 expression within IL-10-producing cells

**Fig. 3:**
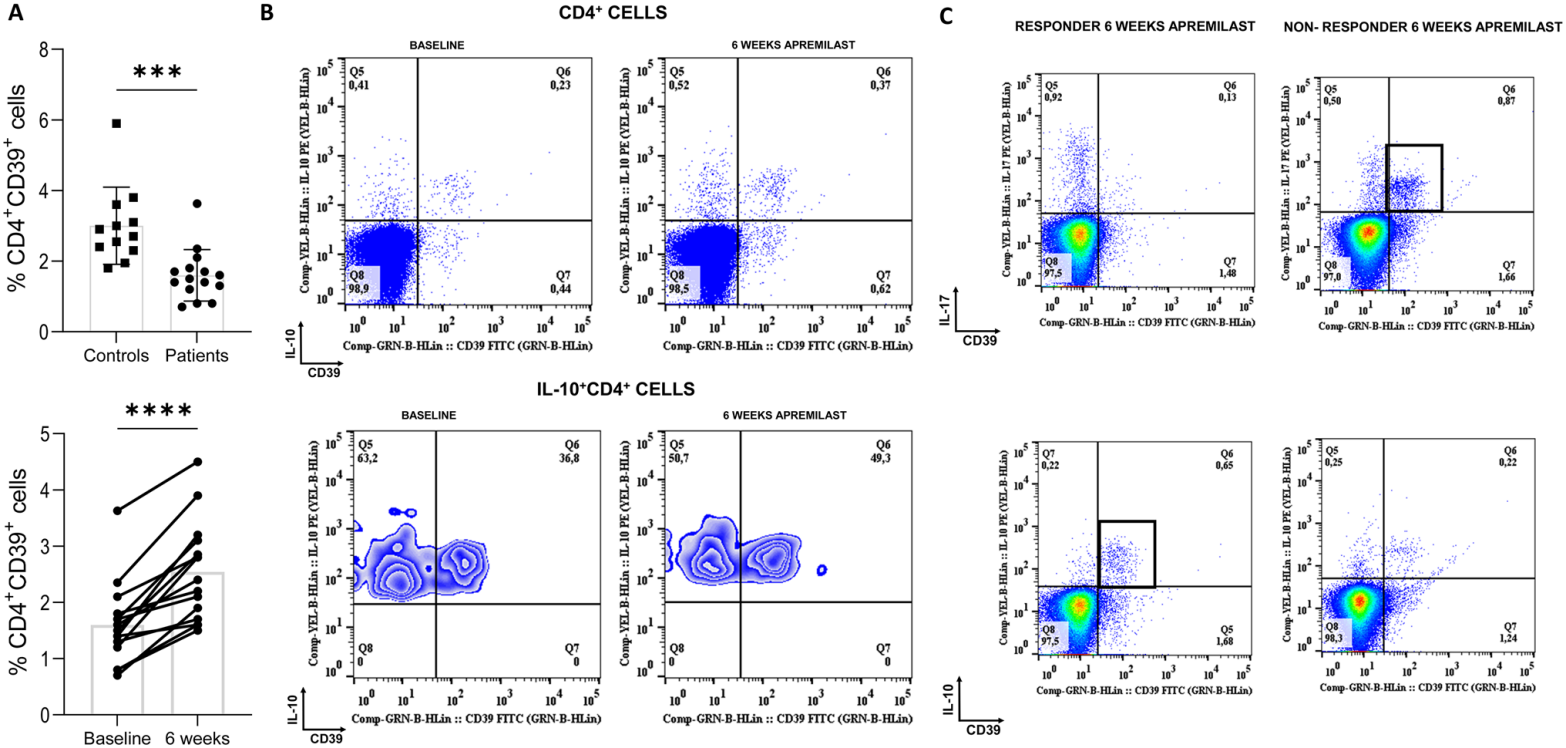
CD39 expression was associated with response to apremilast and interleukin (IL-)10 production.** a** Graphs illustrating that psoriatic patients exhibited decreased levels of CD39 on CD4^+^ T cells compared to controls at baseline, and that apremilast increased CD39 in CD4^+^ T cells. **b** A post-treatment enrichment of CD39 within the IL-10 producing CD4^+^ T cell sub-population was observed. **c** Finally, in responders CD39^+^ cells produced high levels of IL-10, while in non-responders CD39^+^ cells produced IL-17. Unpaired and paired t-tests were executed. A p-score < 0.05 was considered significant. Graphs are depicting mean ± SD; *** p ≤ 0.001; **** p ≤ 0.0001

We also examined CD39^+^ T cells. At baseline, CD39^+^CD4^+^T cells were decreased in PsD patients compared to controls (− 1.40, 95% CI − 2.12 to − 0.68, p = 0.0005). At 6 weeks, apremilast increased CD39^+^CD4^+^T cells (median pair differences [IQR] − 0.95 [− 1.38 to − 0.55], Hodges-Lehmann 95% CI − 1.25 to − 0.60, p = 0.00073, r = 0.880, Fig. 1A) and up to 50% of CD4^+^IL-10^+^ T cells were CD39^+^, with an enrichment of CD39 expression within the IL-10-producing CD4^+^T cells, indicating that CD39^+^CD4^+^T cells produced the immunoregulatory IL-10 (Fig. 3). Interestingly, in responders, CD39^+^ cells produced IL-10, while in non-responders, CD39^+^cells produced IL-17 (Fig. 3). However, a significant fraction of IL-10-producing CD39^+^ cells were non-CD4^+^T cells, and most non-CD4^+^T cells expressing CD39 were CD56^−^CD11c^−^ cells (Supplementary Fig. 1).

## Discussion

Our results show that apremilast inhibits the production of pro-inflammatory cytokines IL-17, IFNγ, and IL-6, and increases the production of immunoregulatory IL-10, in PsD as early as at 6 weeks pot-treatment. The obtained data also provide indirect evidence that such effects are likely mediated in part via the expression of CD39. These preliminary findings support a novel role for apremilast in inducing disease remission in patients with PsD.

The disrupted equilibrium between Tregs and T effector cells in autoimmune disease has been widely reported [18–20]. Our study found that the percentage of CD4^+^CD39^+^ cells is significantly lower in PsD patients than in controls. CD4^+^CD39^+^ cells have been reported to be potent inhibitors of lymphocyte proliferation and prevented CD4^+^CD25^−^ cell expansion, in contrast to CD4^+^CD39^−^ cells[11], suggesting that the acquisition of CD39 positivity in CD4 T cells makes them potent immunosuppressive lymphocytes. Published evidence supports the immunomodulatory role of CD39^+^ cells in autoimmune diseases. For instance, decreased levels of CD39^+^ FoxP3^+^ Tregs have been shown to be impaired in MS [12, 14], whereas during remission, CD39^+^Tregs expand to counter-balance Th17 cell activity[21]. Furthermore, a common single nucleotide polymorphism associated with low levels of CD39^+^ cells has been found to confer increased susceptibility to Crohn's disease [22]. Decreased CD39 expression on Treg cells has been reported in patients with psoriasis compared to controls[23]. In line with these findings, we found decreased CD4^+^CD39^+^cells in PsD, and furthermore, we found that apremilast treatment increased the proportion of CD4^+^CD39^+^ cells.

We also looked at possible mechanisms for the immunoregulatory role of CD39, investigating the association between CD39 and IL-10 expression. A significant fraction of cells within the CD4^+^IL-10^+^ compartment was CD39^+^. The CD39^+^ subset produced a large proportion of IL-10. In human B cells, CD39 expression most frequently was associated with IL-10 production, and its expression was upregulated on IL-10^+^ compared to IL-10^−^ B cells[24]. Interestingly, in our study we observed a post-apremilast treatment enrichment of CD39 expression within the IL-10-producing CD4^+^ T cell sub-population.

The concept of a disease-modifying anti-rheumatic drugs (DMARDs)-mediated effect on the function of Tregs is not unknown[25]. Although our results indicate that apremilast at 6 weeks did not significantly affect Tregs, apremilast mediated an increase of CD4^+^FoxP3^+^cells. This observation may be unsurprising since apremilast was observed to increase CD39^+^CD4^+^T cells and CD39 expression has been found to correlate with FoxP3 expression [11, 26].

Finally, the results of this exploratory pilot study implying that CD39^+^ expression may potentially indicate an apremilast-mediated clinical improvement, are in line with other studies showing that, CD39^+^ Tregs were associated with methotrexate responsiveness in patients with rheumatoid arthritis [27] and that higher CD39^+^ cell frequencies have been associated with responsiveness to methotrexate in patients with this disease [28]. Furthermore, the central role of the adenosine pathway in inflammatory arthritis has also been suggested previously[29].

A limitation of our preliminary study is the small number of patients. Furthermore, no long-term follow-up results were available, and this must be underlined. The limited sample size and the retrospective nature of the study precluded formal power analysis, thus the results should be interpreted as hypothesis-generating, which means that the results must be viewed with caution until confirmed in larger studies. Our results may have also been affected by the sex imbalance of the participating cohort, which represents a limitation inherent to the biobank-based design. Nevertheless, our study shows that apremilast withing 6 weeks increased CD4^+^FoxP3^+^ and CD4^+^CD39^+^cells, decreased in pro-inflammatory cytokines IL-17 and IFNγ, and increased the immunomodulatory cytokine IL-10. The level of FoxP3 expression in T cells, although not significantly different between PsD patients and healthy controls has been linked with autoantibodies in PsD. Intermediate and high FoxP3 expression in Tregs were found to correlate positively and negatively, respectively, with anti-ADAMTSL5 (a disintegrin and metalloproteinase domain containing thrombospondin type 1 motif-like 5) autoantibodies in PsD [30]. Future research in large prospective studies investigating the mechanisms of action of CD39^+^ Tregs in PsD is urgently warranted.

## Supplementary Information

Below is the link to the electronic supplementary material.

Supplementary file1 (PPTX 132 KB)

Supplementary file2 (DOCX 34 KB)

## Data Availability

The corresponding author will provide presented data upon request.
