# Supplementary figures and images for: Apremilast therapy increases CD39+CD4+ T cells in peripheral blood of patients with psoriatic disease: a retrospective observational pilot study
Source: Rheumatol Int. 2026 May 6;46(5):84. doi: 10.1007/s00296-026-06114-3 (PMC13149556; doi:10.1007/s00296-026-06114-3)

## Slide 1
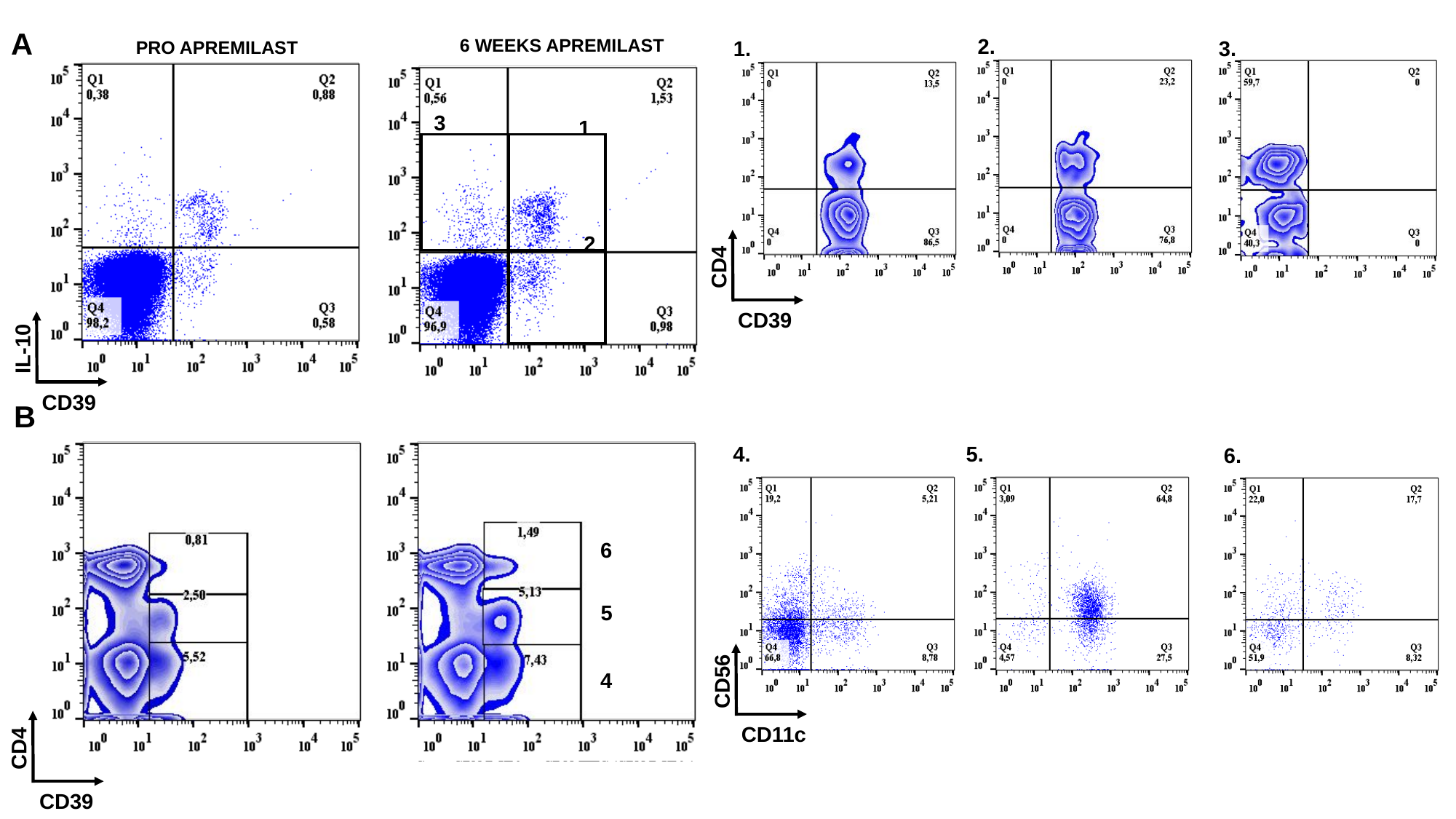

A
2.
6 WEEKS APREMILAST
3.
1.
PRO APREMILAST
3
1
2
CD4
CD39
IL-10
CD39
B
4.
5.
6.
6
5
CD56
CD11c
4
CD4
CD39

Supplement: Supplementary file 1 — Supplementary file1 (PPTX 132 KB) [file 296_2026_6114_MOESM1_ESM.pptx]
